# Do School-Level Factors Affect the Health Behaviors of High School Students in Korea?

**DOI:** 10.3390/ijerph19020751

**Published:** 2022-01-10

**Authors:** Seon-Hui Kwak, Hyo-Jin Lee, Bo-Mi Shin

**Affiliations:** 1 Department of Dental Hygiene, College of Dentistry, Gangneung Wonju National University, Gangneung-si 25457, Korea; tjsgml0617@gwnu.ac.kr (S.-H.K.); leehjin@gwnu.ac.kr (H.-J.L.); 2 Research Institute of Oral Science, Gangneung Wonju National University, Gangneung-si 25457, Korea

**Keywords:** adolescent health, health behavior, multilevel analysis, school-based health services, social health determinants

## Abstract

We conducted a multilevel analysis to identify the individual- and school-level factors that affect Korean high school students’ tooth brushing, soda intake, smoking, and high-intensity physical activity. We sampled 27,919 high school students from the 15th Korea Youth Risk Behavior Web-Based Survey. The individual-level variables included demographic, socioeconomic, and health-related factors. The school-level variables included school system and school type. Regarding the individual-level factors, economic level and academic performance had a significant effect on health behavior when the demographic variables were adjusted. In the final model, the school-level factors had a significant effect on health behavior. The odds ratio (OR) of brushing less than twice a day in vocational schools compared to general schools was 1.63 (*p* < 0.001), and the OR of soda intake more than three times a week in vocational schools was 1.33 (*p* < 0.001). In addition, the OR of smoking in vocational schools was 2.89 (*p* < 0.001), and the OR of high-intensity physical activity in vocational schools was 0.80 (*p* < 0.001). Therefore, both individual- and school-level factors affect Korean students’ health behaviors. A school-based comprehensive health promotion strategy should be developed that considers schools’ characteristics to equip all students with health awareness, regardless of socioeconomic status.

## 1. Introduction

Global interest in non-communicable diseases (NCDs), such as cardiovascular and respiratory diseases, is increasing. NCDs are caused by the complex interaction of genetic, physiological, environmental, and behavioral factors. More than 2.1 billion children were affected by NCDs in 2017, more than two-thirds of those with a history of an NCD experiencing onset during adolescence, thus increasing the burden of NCDs among adolescents [1]. NCDs are caused by risk factors such as smoking, unhealthy diet, physical inactivity, and harmful alcohol consumption, which start during adolescence [2,3]. These behaviors increase young people’s risk of NCDs [1]. The Centers for Disease Control and Prevention (CDC) reported that 31.6% of adolescents had a body mass index (BMI) of 25 or higher in 2019; this figure has increased annually since 1999 [4]. Moreover, the percentage of high school students who were physically active for a total of ≥60 min/day on all seven days decreased from 28.7% in 2011 to 23.2% in 2019, and the percentage of those who ate vegetables <1 time/day increased from 37.7% in 2011 to 40.7% in 2019 [5]. The National Survey on Drug Use and Health in the United States (NSDUH) also reported that the percentage of American adolescents who were experiencing depression increased from 8.8% in 2005 to 15.7% in 2019 [6].

These trends are similar to the health status of Korean adolescents. The 15th Korea Youth Risk Behavior Survey (KYRBS), which investigates the health behavior of Korean adolescents [7], showed that the percentage of adolescents who had a BMI of 25 or higher increased from 12.1% in 2009 to 20.7% in 2019. Moreover, the percentage of those who were physically active for a total of ≥60 min/day on all seven days increased from 13.1% in 2016 to 14.7% in 2019, and the percentage of those who ate vegetables ≤1 time/day increased from 57.5% in 2016 to 62.2% in 2019. The percentage of those experiencing depression increased from 25.5% in 2016 to 28.2% in 2019 [7].

Adolescence denotes the transition period between childhood and adulthood and is an important time for building relationships, developing social skills, and learning lifelong health behaviors [8]. An individual’s health behavior is affected by the various social determinants of health that surround them. Therefore, factors such as income, education, unemployment, working and living conditions, access to medical services, and school, community, and national policies can affect an individual’s health. However, previous studies have revealed that mainly individual-level factors, such as parental income level, education level, parental health behavior, and health perception, influence adolescents’ health behaviors such as smoking, diet, physical activity, and oral hygiene care [9,10,11,12].

Recently, it has been suggested that proximal or intermediate determinants influence adolescents’ health behaviors at both the social-level and individual level [8]. The World Health Organization’s (WHO) Commission on the Social Determinants of Health [13] states that health inequalities can affect health outcomes depending on the environment of growth during early infancy and childhood, the school environment, the work environment, and the community environment, emphasizing the need to improve individuals’ daily living environments to reduce health inequality. Schools constitute the best health settings as they can provide a supportive environment for all students, operate prevention-focused health promotion programs, and control risk factors such as sugar intake, alcohol consumption, and smoking [14]. Adolescents spend much of their time at school and can easily participate in health-related education programs run by the school [15]. Therefore, efforts should be made to improve adolescents’ daily living environments, eliminate risk factors, and strengthen protective factors by implementing a school-centered comprehensive intervention strategy [8].

Efforts to address the risk factors of NCDs can contribute to adolescents’ lifelong health [2]. Therefore, it is necessary to identify the factors that influence the formation of health behaviors from multilateral aspects and to apply preventive interventions and strategies [16] to reduce adolescents’ exposure to risk factors. Previous studies have reported that adolescent smoking is strongly influenced by their peers’ behavior in the school setting [17,18]. Meanwhile, a previous study on adolescents’ diets revealed that when schools push policies such as limiting the sale of soda and instead provide healthy alternatives (e.g., low-fat milk or water), the consumption of sweetened beverages decreased [19]. Previous studies have shown that school policies and teacher interventions affected physical activity and toothbrushing performance, thus highlighting how influential the role of school environments is [20]. However, there is insufficient research on the influence of schools on the health risk behaviors that cause NCDs in adolescents in Korea. Therefore, this study attempted to examine individual-level and school-level factors that affect Korean adolescents’ smoking, diet, physical activity, and oral health behaviors. We conducted a multilevel analysis using the KYRBS national statistical data, as it can represent all adolescents in Korea.

## 2. Method

### 2.1. Study Population

This study used the 15th KYRBS (2019) to perform a secondary data analysis and identify the individual-level and school-level factors affecting high school students’ smoking, diet, physical activity, and oral health behaviors [7]. The KYRBS is a government-approved nationwide statistical survey on students who are enrolled at the middle school and high school levels in Korea (approval number: 117058). It identifies the current statuses and trends of adolescents’ health behaviors in Korea across 15 categories, which include smoking, alcohol consumption, physical activity, and oral health behavior, among others. The KYRBS was designed with stratified and multistage cluster sampling that considered regions and schools to minimize sample errors. During the primary extraction, samples of 400 middle schools and 400 high schools were selected from 17 cities and provinces in Korea using permanent random number extraction. During the secondary sampling, one class was randomly selected by grade from the selected sample schools. High schools were proportionally extracted according to school type and school system so that the population and sample composition ratios were consistent. All students in the selected sample class used school computers to access the KYRBS website with the participation number. After reading the participation agreement provided on the website and consenting to participate, they pressed the start button to begin the KYRBS anonymously. A total of 57,303 students participated in the survey (participation rate: 95.3%). In order to consider the various characteristics of schools, this study selected 27,919 high school students from the first to third grades as the final subjects, excluding middle school students who received the same education without dividing the school system.

### 2.2. Dependent Variables

Oral health behavior, diet, smoking, and physical activity are representative risk factors for systemic and oral diseases [14,21]. Since this study attempted to identify the factors that affect Korean adolescents’ health behavior performance, the following four behaviors were selected from among the KYRBS questions as the dependent variables: “tooth brushing less than twice a day”, “soda intake more than three times a week”, “smoking” and “performing high-intensity physical activities less than two days a week”.

Regarding oral health behavior, the KYRBS’s questions included, “How many times did you brush your teeth yesterday?” If the response was “zero–two times”, then we defined the result as “tooth brushing less than twice a day” and if the response was “three–nine times or more”, then the variable was defined as “tooth brushing more than three times a day”.

Regarding diet, the KYRBS’s questions included, “How often have you drunk soda (excluding carbonated water) in the last seven days?” If the responses ranged from “three-four times a week” to “more than three times a day”, then the variables were defined as “soda intake more than three times a week”.

Regarding smoking, the KYRBS’s questions included, “How many days did you smoke, including even a single regular cigarette, in the last 30 days?” If the responses were “not applicable”, or “none in the last 30 days”, then the variable was defined as “non-smoking”. If the response was “one or two days a month” to “every day”, then the variable was defined as “currently smoking”.

Regarding physical activity, the KYRBS’s questions included, “In the last seven days, how many days did you do 20 min or more of high-intensity physical activity that made you short of breath or sweat?” Responses of “none in the last seven days”, “one day a week”, or “two days a week”, were defined as “high-intensity physical activity less than two days a week”. When the responses were “three days a week” to “five days a week or more”, the variable was defined as “high-intensity physical activity more than three days a week”.

### 2.3. Individual-Level Variables

The individual-level independent variables were derived from previous studies on factors affecting adolescents’ health behavior [22,23,24] and were classified as demographic, socioeconomic, and health-related factors, including self-perceived health conditions and psychological factors. Demographic factors included sex and grade. Grades reflect students’ age; the first grade contained students aged 16 years old, the second grade contained students aged 17 years old, and the third grade contained students aged 18 years old.

Socioeconomic factors included self-reported economic status and academic performance. To determine students’ self-reported economic status, they were asked: “What is the economic status of your family?” Responses of “high” or “slightly high” placed students in the “high” category, responses of “middle” placed students in the “middle” category, and responses of “lower-middle” or “low” placed students in the “low” category. Students were asked about their academic performance in the last 12 months, and “high” or “slightly high” responses placed them in the “high” category, “middle” responses placed them in the “middle” category, and “lower-middle” or “low” responses placed them in the “low” category.

Health health-related factors included self-reported health status, self-reported oral health status, experience of stress, experience of depression, and experience of oral symptoms. Regarding the self-reported health and oral health statuses, the students were asked: “How do you feel about your health in general?” and “How do you usually feel about your oral health, such as teeth and gums?” If they responded, “I am very healthy” or “I am in good health”, then they were categorized as “healthy”. Responses of “normal”, “not in good health” or “very unhealthy” categorized students as “unhealthy”.

Regarding stress, students were asked: “How often do you feel stressed?” If the students responded “often” or “a lot”, then they were categorized as having experienced stress (i.e., “yes”). Responses of “sometimes”, “not often”, or “not at all”, then then they were categorized as not having experienced stress (i.e., “no”). Regarding depression, students were asked: “During the past 12 months, have you ever felt so sad or hopeless that you stopped your daily routine for two weeks?” The results of the “yes” and “no” responses were used as-is.

Regarding the experience of oral symptoms, students were asked if they had symptoms related to six oral diseases (e.g., toothache, gingival bleeding, gum pain, and bad breath) in the last 12 months. If the value was “zero” after summarizing all experiences for each symptom, then the students were categorized as having no experience (i.e., “no”), and if the value was “one–six”, then the students were categorized as having experience (i.e., “yes”).

### 2.4. School-Level Variables

Regarding the school-level independent variables, we used the school system and school type variables from the raw data of the 15th KYRBS. In the KYRBS, the school system is divided into two categories: general high school and vocational high school. The school type is divided into three categories: boys’ school, girls’ school, and co-ed school. General high schools provide a broad education in a variety of fields rather than a specific field. Vocational high schools specialize in experiential education such as field training that aims to nurture talent in a specific field for students with similar aptitudes and abilities [25,26]. Vocational high schools focus students on getting a job after graduation rather than going to college. The school type variable was composed of three categories and analyzed by converting it into a dummy variable.

### 2.5. Statistical Analysis

A two-level analysis was performed to identify the factors at the individual- and school-levels that affected Korean adolescents’ health behaviors. Since the dependent variables in this study were dichotomous variables, they had a Bernoulli distribution; thus, multilevel logistic regression analysis was performed to transform the probabilities into logit values, which were expressed as a linear relationship. Regarding the independent variables, the individual-level factor variables were used in one-level units, and the school-level factor variables were used in two-level units. As a result of confirming the multicollinearity of the independent variables at the individual and school level, the variance inflation factor (VIF) value was found to be in the range of 1.006 to 1.204. As the VIF value was less than 10, it was confirmed that there was no correlation between the independent variables.

For the statistical analysis, we set the dependent variables as tooth brushing less than twice a day, soda intake more than three times a week, current smoking, and high-intensity physical activity less than two days a week. The analysis was performed in three steps: a null model, an unconditional slope model, and a conditional model. The null model checked whether the dependent variables differed between schools through an intraclass correlation coefficient (ICC). Since the dependent variables were dichotomous variables, the ICC was calculated as follows:

ICC = τ_00_(2 − level variance)/[τ_00_(2 − level variance) + (π^2^/3)] (1)

The unconditional slope model checked the random and fixed effects and significant individual-level factors by inputting the individual-level independent variables into the null model. The conditional model confirmed which individual- and school-level independent variables had a significant effect on the dependent variables by adding the school-level independent variables to the existing model. We conducted a likelihood ratio test to test the fit of the model. Deviance was confirmed by using −2LL (log likelihood) using the maximum likelihood method. Statistical analysis was performed using SPSS 23.0 (SPSS Inc., Chicago, IL, USA) and HLM 7.0 (Scientific Software International Inc., Chicago, IL, USA).

## 3. Results

### 3.1. Health Behaviors according to the Students’ General Characteristics

Table 1 shows the general characteristics of the students’ health behavior practice rates. The tooth brushing rate of boys was lower than that of girls, and the rate of soda intake and current smoking of boys was higher than that of girls (*p* < 0.001). The lower the self-reported economic status, the lower the tooth brushing rate and the higher the smoking rate (*p* < 0.001). Soda intake was not significantly different according to economic status (*p* = 0.448). Also, the lower the academic performance, the lower the rate of tooth brushing and high-intensity physical activity, and the higher the soda intake and smoking (*p* < 0.001).

### 3.2. Individual-Level Factors Affecting Students’ Health Behaviors

Table 2 shows the results of the individual-level factors that affect students’ health behaviors after adjusting for demographic factors. The self-reported economic status and academic performance were found to have a significant association with all health behaviors. The adjusted odds ratio (OR) of tooth brushing less than twice a day for students with a lower economic status was 1.27 (confidence interval (CI = 1.18−1.37; *p* < 0.001). The OR of soda intake more than three times a week for students with lower academic grades was 1.39 (CI = 1.30−1.48; *p* < 0.001). The OR of current smoking for students with lower academic grades was 2.29 (CI = 2.06−2.55; *p* < 0.001).

### 3.3. School-Level Factors Affecting Students’ Health Behaviors

The school-level factors that affected high school students’ health behavior varied between the school system and type. Table 3 shows that the OR of brushing less than twice a day in vocational high schools compared to general high schools was 1.63 (CI = 1.43–1.87; *p* < 0.001); in boys’ schools compared to co-ed schools, 1.40 (CI = 1.26–1.56; *p* < 0.001); and in girls’ schools compared to co-ed schools, 1.22 (CI = 1.05–1.43; *p* = 0.010).

Table 4 shows that the OR of soda intake more than three times a week in vocational high schools compared to general high schools was 1.33 (CI = 1.21–1.46; *p* < 0.001), and 0.88 (CI = 0.80–0.98; *p* = 0.019) for girls’ schools compared to co-ed schools.

Table 5 shows that the OR of current smoking in vocational high schools compared to general high schools was 2.89 (CI = 2.48–3.38; *p* < 0.001), and 0.65 (CI = 0.51–0.82; *p* < 0.001) for girls’ schools compared to co-ed schools.

Table 6 shows that the OR of high-intensity physical activity less than two days a week in vocational high schools compared to general high schools was 0.80 (CI = 0.72–0.89; *p* < 0.001), and 1.14 (CI = 1.03–1.26; *p* = 0.013) for boys’ schools compared to co-ed schools.

## 4. Discussion

Adolescence is an important period for the development of health and disease protection. Practicing unhealthy behaviors during adolescence increases the risk of exposure to NCDs and the risk of disease incidence in adulthood and old age. Since health risk behaviors can cause chronic NCDs such as cardiovascular disease and cancer, it is important to establish correct health behaviors during adolescence [27]. Therefore, this study conducted a multilevel analysis using representative national statistical data from the KYRBS to identify the individual- and school-level factors that affect the health behavior of Korean high school students.

The results showed that socioeconomic factors, such as economic status and academic performance, affected tooth brushing, soda intake, smoking, and physical activity. This is consistent with the previous studies that have confirmed the importance of socioeconomic factors at the individual level as vital health determinants to assess adolescents’ health behavior [23,28]. Beaglehole et al. [29] found that smoking, diet, alcohol consumption, and physical activity (i.e., the causes of NCDs) were unequally managed depending on income level, and that those who received lower incomes did not receive comprehensive prevention and treatment of either NCDs or the risk factors due to financial reasons and poor local healthcare systems. To reduce such inequality, these authors advocated for a more practical and cost-effective strategy for the whole population. Socio-economic inequality also affects the health behaviors of Korean adolescents. Therefore, in order to reduce this inequality, it is necessary to prepare a strategy that enables all adolescents to manage health risk factors including oral hygiene, diet, smoking, and physical activity in an integrated manner, based on the population approach.

Our results confirmed that the four health risk behaviors of Korean high school students have a significant effect on the school system among the school-level factors. The OR of the vocational high school students for brushing less than twice a day was 1.63 times higher than that of the general high school students. Moreover, their soda intake was 1.33 times higher, and their smoking rate was 2.89 times higher. Finally, the vocational high school students had a higher physical activity practice rate. These results are consistent with those of the previous studies that have confirmed a significant difference in health behaviors according to the high school system [30,31]. According to Atorkey et al.’s [32] systematic review, as vocational high school students become more independent during vocational education training and tend to choose their own lifestyle habits, they have an increased risk of engaging in unhealthy behaviors such as smoking, eating fast food, and physical inactivity. Since the risk factors affecting these health behaviors appear complex, a comprehensive and preventive intervention strategy should be developed and delivered in the school environment to promote the health of vocational high school students. A comprehensive school-centered intervention strategy that considers the type of schools is needed to improve the health behaviors and reduce the risk factors of Korean high school students.

Schools can connect education with health and provide equal education to all students. The WHO considers schools to be effective platforms for implementing comprehensive practices that provide the necessary life skills for NCD prevention and has proposed the Health Promotion School (HPS) model [33]. The HPS is a school-based strategy that empowers students with health awareness, via education and practice, and builds a supportive environment that enables them to adopt life skills so that they can live healthy lives. When operating the HPS using secondary school students in the Netherlands, Busch et al. [34] found that the provision of theoretical and practical education on health behaviors, school-based comprehensive interventions, and student-led policies (e.g., creating healthy school food in the cafeteria, alcohol-free school parties, and establishing smoke-free zones) resulted in improved student health behaviors.

Similarly, in Korea, to improve students’ health and the health risk behaviors that cause NCDs, some schools have operated the HPS since 2009 and have integrated multiple disciplines such as tobacco cessation, nutrition, physical activity, oral health, and mental health. However, as of 2019, only around 0.4% of all 11,657 elementary, middle, and high schools in Korea were operating the HPS, and 53.0% of those participating schools were elementary schools. In addition, the HPS has been evaluated as insufficient because its limited operation mainly focuses on physical activity among various health areas [35]. This indicates that there is little interest in school-based integrated strategies for adolescents’ health problems in Korea, and it is estimated that this is because a social consensus has not yet been reached to activate it. However, school-based comprehensive interventions that provide health intervention strategies to all students, regardless of socioeconomic inequality, have a positive effect on improving adolescents’ health behavior [15,36]. Therefore, to improve Korean adolescents’ health behavior and health promotion, it is necessary to develop an integrated health program in schools based on the HPS model and to actively utilize this program by applying it to middle schools and high schools.

An important way to prevent NCDs is to reduce disease-related common risk factors in adolescents and promote interventions by collaborating with multisectoral stakeholders, including governments and relevant associations, schools, and communities. For example, Canada has established comprehensive health policies and guidelines that range from simple nutrition guidelines (e.g., no candy is to be given to students as a reward) to policies (e.g., daily physical activity is to be practiced from kindergarten through to 12th grade) to operate the Comprehensive School Health (CSH) model. Further, Canada uses the CSH model to continuously monitor the improvements in students’ knowledge, attitudes, and behaviors [37]. Therefore, the Korean Ministry of Education and other related government agencies, such as the Ministry of Health and Welfare, should prepare school-based integrated health promotion policies and guidelines based on foreign cases and should establish a monitoring system to activate health programs in schools that can address modifiable risk factors of NCDs such as smoking, physical activity, oral hygiene, and diet.

This study attempted to increase the possibility of generalization by analyzing nationally approved statistical data that are representative of high school students in Korea. However, some limitations remain. First, confirming the causal relationship between risk factors and health behaviors by analyzing data based on cross-sectional studies was limited. Second, we were unable to analyze the variables of various characteristics (e.g., school policy, the number of school teachers, school-based health education, or health program practice rate) because secondary data were used. Despite these limitations, this study was meaningful in that it identified individual-level factors and school-level factors that affect the health behaviors of Korean high school students using large-scale data from a national sample.

## 5. Conclusions

According to the results of this study, not only individual-level factors but school-level factors (e.g., school system and school type) also have a significant effect on the health behavior of high school students in Korea. Because school-based health programs could provide opportunities for the health promotion of all students, regardless of their socioeconomic status, it is necessary to develop comprehensive school-based health promotion strategies based on the characteristics of schools.

## Figures and Tables

**Table 1:** Health behaviors according to the students’ general characteristics.

Variable		Total	Tooth Brushing Less than Twice a Day	*p*-Value	Soda Intake More than Three Times a Week	*p*-Value	Current Smoking	*p*-Value	High-Intensity Physical Activity Less than Two Days a Week	*p*-Value
		N (wt%)	N (wt%)		N (wt%)		N (wt%)		N (wt%)	
	Total	27,919 (100.0)	12,859 (46.4)		10,376 (37.4)		2774 (9.9)		20,655 (73.9)	
Demographic factors	Sex									
	Boy	14,440 (52.5)	7642 (53.1)	<0.001	6581 (45.9)	<0.001	2041 (14.2)	<0.001	8889 (61.6)	<0.001
	Girl	13,479 (47.8)	5217 (39.0)		3795 (28.2)		733 (5.2)		11,766 (87.2)	
	Grade									
	1st	9273 (32.9)	4473 (49.1)	<0.001	3380 (36.7)	0.235	728 (8.3)	<0.001	6803 (73.3)	<0.001
	2nd	9044 (31.6)	4245 (47.1)		3373 (38.0)		926 (10.0)		6625 (72.5)	
	3rd	9602 (35.6)	4141 (43.2)		3623 (37.6)		1120 (11.4)		7227 (75.6)	
Socioeconomic factors	Self-reported economic status									
	Low	4432 (15.4)	2304 (52.4)	<0.001	1682 (38.3)	0.448	589 (12.6)	<0.001	3293 (73.9)	<0.001
	Middle	13,937 (50.0)	6438 (46.4)		5163 (37.3)		1242 (9.1)		10,528 (75.5)	
	High	9550 (34.6)	4117 (43.6)		3531 (37.3)		943 (9.9)		6834 (71.5)	
	Academic performance									
	Low	10,012 (36.0)	4990 (50.4)	<0.001	4107 (41.2)	< 0.001	1434 (14.3)	<0.001	7198 (71.8)	<0.001
	Middle	8827 (31.7)	3862 (43.8)		3210 (36.8)		695 (7.8)		6656 (75.1)	
	High	9080 (32.3)	4007 (44.4)		3059 (33.9)		645 (7.1)		6801 (74.9)	
Health-related factors	Self-reported health status									
	Unhealthy	9367 (33.5)	4508 (48.4)	<0.001	3554 (38.3)	0.035	904 (9.6)	0.124	7918 (84.4)	<0.001
	Healthy	18,552 (66.5)	8353 (45.4)		6822 (37.0)		1870 (10.1)		12,737 (68.6)	
	Self-reported oral health status									
	Unhealthy	19,599 (70.2)	9714 (49.9)	<0.001	7534 (38.8)	< 0.001	1928 (9.8)	0.468	14,837 (75.6)	<0.001
	Healthy	8320 (29.8)	3145 (38.0)		2842 (34.3)		846 (10.1)		5818 (69.8)	
	Experience of stress									
	Yes	11,935 (42.4)	5414 (45.7)	0.062	4498 (38.0)	0.103	1313 (11.1)	<0.001	9309 (78.0)	<0.001
	No	15,984 (57.6)	7445 (46.9)		5878 (37.0)		1461 (9.1)		11,346 (70.8)	
	Experience of depression									
	Yes	8226 (29.4)	3660 (44.9)	0.001	3217 (31.1)	<0.001	1149 (13.9)	<0.001	6167 (74.6)	0.088
	No	19,693 (70.6)	9199 (47.0)		7159 (36.5)		1625 (8.3)		14,488 (73.6)	
	Oral symptoms									
	Yes	16,984 (60.8)	8211 (48.7)	<0.001	6402 (38.2)	0.001	1701 (10.0)	0.562	12,984 (76.4)	<0.001
	No	10,935 (39.2)	4648 (42.8)		3974 (36.2)		1073 (9.8)		7671 (69.9)	

The data were analyzed by complex sample analysis.

**Table 2:** Individual-level factors affecting students’ health behaviors.

	Variable	Tooth Brushing Less than Twice a Day		Soda Intake More than Three Times a Week		Current Smoking		High-Intensity Physical Activity Less than Two Days a Week	
		OR (95%CI)	*p*	OR (95%CI)	*p*	OR (95%CI)	*p*	OR (95%CI)	*p*
Demographic factors	Sex								
	Boy	1.86 (1.71–2.03)	<0.001	2.29 (2.14–2.44)	<0.001	3.38 (2.91–3.92)	<0.001	0.26 (0.24–0.28)	<0.001
	Girl	1.00		1.00		1.00		1.00	
	Grade								
	1st	1.31 (1.21–1.41)	<0.001	0.97 (0.91–1.03)	0.264	0.70 (0.62–0.79)	<0.001	0.92 (0.84–1.00)	0.046
	2nd	1.18 (1.10–1.27)	<0.001	1.01 (0.95–1.08)	0.705	0.86 (0.76–0.97)	0.012	0.85 (0.78–0.93)	<0.001
	3rd	1.00		1.00		1.00		1.00	
Socioeconomic factors	Self-reported economic status								
	Low	1.27 (1.18–1.37)	<0.001	0.92 (0.85–0.99)	0.025	1.00 (0.88–1.13)	0.963	1.04 (0.95–1.13)	0.454
	Middle	1.06 (1.01–1.12)	0.027	0.95 (0.90–1.01)	0.098	0.85 (0.77–0.93)	0.001	1.16 (1.09–1.24)	<0.001
	High	1.00		1.00		1.00		1.00	
	Academic performance								
	Low	1.19 (1.12–1.27)	<0.001	1.39 (1.30–1.48)	<0.001	2.29 (2.06–2.55)	<0.001	0.75 (0.69–0.80)	<0.001
	Middle	0.97 (0.91–1.03)	0.318	1.19 (1.12–1.27)	<0.001	1.21 (1.07–1.36)	0.002	0.91 (0.84–0.98)	0.016
	High	1.00		1.00		1.00		1.00	
Health-related factors	Self-reported health status								
	Unhealthy	-	-	-	-	-	-	2.09 (1.94–2.25)	<0.001
	Healthy	-		-		-		1.00	
	Self-reported oral health status								
	Unhealthy	1.59 (1.51–1.68)	<0.001	1.22 (1.15–1.29)	<0.001	-	-	-	-
	Healthy	1.00		1.00		-		-	
	Experience of stress								
	Yes	-	-	-	-	-	-	1.01 (0.95–1.08)	0.782
	No	-		-		-		1.00	
	Experience of depression								
	Yes	0.93 (0.88–0.98)	0.005	1.24 (1.17–1.31)	<0.001	2.01 (1.83–2.20)	<0.001	-	-
	No	1.00		1.00		1.00		-	
	Oral symptoms								
	Yes	1.25 (1.19–1.30)	<0.001	1.12 (1.06–1.18)	<0.001	-	-	1.14 (1.08–1.20)	<0.001
	No	1.00		1.00		-		1.00	

The data were analyzed by complex sample logistic regression. Values are presented as odds ratio (95% confidence interval). OR, odds ratio; CI, confidence interval.

**Table 3:** Multilevel analysis of students who brush twice a day or less.

Model Parameter		Model 1 Null Model		Model 2 Unconditional Slope Model		Model 3 Final Model	
		OR (95%CI)	*p*	OR (95%CI)	*p*	OR (95%CI)	*p*
Fixed effect							
Level 1	Intercept γ_00_	0.87 (0.82–0.93)	<0.001	0.33 (0.29–0.37)	<0.001	0.26 (0.23–0.30)	<0.001
	Sex (boy vs. girl)			1.74 (1.60–1.88)	<0.001	1.72 (1.57–1.89)	<0.001
	Grade (1st vs. 3rd)			1.28 (1.19–1.37)	<0.001	1.28 (1.19–1.38)	<0.001
	Grade (2nd vs. 3rd)			1.17 (1.09–1.26)	<0.001	1.18 (1.10–1.26)	<0.001
	Self-reported economic status (lower vs. upper)			1.24 (1.15–1.33)	<0.001	1.22 (1.14–1.32)	<0.001
	Self-reported economic status (middle vs. upper)			1.10 (1.04–1.16)	<0.001	1.09 (1.03–1.15)	0.003
	Academic performance (lower vs. upper)			1.19 (1.12–1.27)	<0.001	1.20 (1.13–1.28)	<0.001
	Academic performance (middle vs. upper)			1.00 (0.94–1.06)	0.914	1.00 (0.94–1.07)	0.985
	Self-reported health status (unhealthy vs. healthy)			-	-	-	-
	Self-reported oral health status (unhealthy vs. healthy)			1.52 (1.45–1.60)	<0.001	1.53 (1.45–1.61)	<0.001
	Experience of stress (yes vs. no)			-	-	-	-
	Experience of depression (yes vs. no)			0.93 (0.89–0.98)	0.010	0.94 (0.89–0.99)	0.015
	Oral symptoms (yes vs. no)			1.24 (1.19–1.30)	<0.001	1.25 (1.19–1.31)	<0.001
Level 2	School system (vocational vs. general)					1.63 (1.43–1.87)	<0.001
	School type (boys’ vs. co-ed)					1.40 (1.26–1.56)	<0.001
	School type (girls’ vs. co-ed)					1.22 (1.05–1.43)	0.010
Random effect							
School-level variance Tau		0.35	<0.001	0.29	<0.001	0.23	<0.001
ICC		0.10		0.08		0.07	
Deviance		78,247.90		78,172.12		78,142.22	
Reliability		0.844		0.813		0.778	

The data were analyzed by multilevel logistic regression. Values are presented as odds ratio (95% confidence interval). OR, odds ratio; CI, confidence interval.

**Table 4:** Multilevel analysis of students’ soda intake (more than three times a week).

Model Parameter		Model 1 Null Model		Model 2 Unconditional Slope Model		Model 3 Final Model	
		OR (95%CI)	*p*	OR (95%CI)	*p*	OR (95%CI)	*p*
Fixed effect							
Level 1	Intercept γ_00_	0.60 (0.57–0.63)	<0.001	0.28 (0.25–0.31)	<0.001	0.20 (0.18–0.24)	<0.001
	Sex (boy vs. girl)			2.16 (2.02–2.30)	<0.001	2.06 (1.91–2.23)	<0.001
	Grade (1st vs. 3rd)			0.95 (0.89–1.02)	0.171	0.95 (0.89–1.02)	0.174
	Grade (2nd vs. 3rd)			0.99 (0.92–1.06)	0.738	0.99 (0.92–1.06)	0.741
	Self-reported economic status (lower vs. upper)			0.88 (0.81–0.95)	0.002	0.86 (0.79–0.93)	<0.001
	Self-reported economic status (middle vs. upper)			0.93 (0.88–0.99)	0.019	0.93 (0.88–0.98)	0.011
	Academic performance (lower vs. upper)			1.37 (1.29–1.47)	<0.001	1.39 (1.30–1.48)	<0.001
	Academic performance (middle vs. upper)			1.18 (1.10–1.26)	<0.001	1.18 (1.11–1.26)	<0.001
	Self-reported health status (unhealthy vs. healthy)			-	-		
	Self-reported oral health status (unhealthy vs. healthy)			1.22 (1.15–1.29)	<0.001	1.22 (1.15–1.29)	<0.001
	Experience of stress (yes vs. no)			-	-	-	
	Experience of depression (yes vs. no)			1.21 (1.14–1.29)	<0.001	1.22 (1.15–1.29)	<0.001
	Oral symptoms (yes vs. no)			1.11 (1.05–1.17)	<0.001	1.11 (1.05–1.17)	<0.001
Level 2	School system (vocational vs. general)					1.33 (1.21–1.46)	<0.001
	School type (boys’ vs. co-ed)					1.03 (0.95–1.13)	0.479
	School type (girls’ vs. co-ed)					0.88 (0.80–0.98)	0.019
Random effect							
Variance		0.18	<0.001	0.08	<0.001	0.07	<0.001
ICC		0.05		0.02		0.02	
Deviance		78,038.54		77,955.32		77,963.66	
Reliability		0.730		0.549		0.507	

The data were analyzed by multilevel logistic regression. Values are presented as odds ratio (95% confidence interval). OR, odds ratio; CI, confidence interval.

**Table 5:** Multilevel analysis of students’ smoking.

Model Parameter		Model 1 Null Model		Model 2 Unconditional Slope Model		Model 3 Final Model	
		OR (95%CI)	*p*	OR (95%CI)	*p*	OR (95%CI)	*p*
Fixed effect							
Level 1	Intercept γ_00_	0.12 (0.11–0.13)	<0.001	0.04 (0.04–0.05)	<0.001	0.03 (0.03–0.04)	<0.001
	Sex (boy vs. girl)			2.91 (2.54–3.32)	<0.001	2.72 (2.33–3.18)	<0.001
	Grade (1st vs. 3rd)			0.64 (0.57–0.72)	<0.001	0.63 (0.56–0.71)	<0.001
	Grade (2nd vs. 3rd)			0.86 (0.77–0.97)	0.010	0.86 (0.76–0.96)	0.009
	Self-reported economic status (lower vs. upper)			0.90 (0.80–1.01)	0.064	0.86 (0.77–0.97)	0.014
	Self-reported economic status (middle vs. upper)			0.77 (0.71–0.84)	<0.001	0.76 (0.69–0.83)	<0.001
	Academic performance (lower vs. upper)			2.22 (1.99–2.47)	<0.001	2.32 (2.07–2.59)	<0.001
	Academic performance (middle vs. upper)			1.25 (1.13–1.40)	<0.001	1.29 (1.15–1.44)	<0.001
	Self-reported health status (unhealthy vs. healthy)			-	-	-	-
	Self-reported oral health status (unhealthy vs. healthy)			-	-	-	-
	Experience of stress (yes vs. no)			-	-	-	-
	Experience of depression (yes vs. no)			2.01 (1.84–2.19)	<0.001	2.06 (1.88–2.26)	<0.001
	Oral symptoms (yes vs. no)			-	-	-	-
Level 2	School system (vocational vs. general)					2.89 (2.48–3.38)	<0.001
	School type (boys’ vs. co-ed)					0.98 (0.84–1.14)	0.811
	School type (girls’ vs. co-ed)					0.65 (0.51–0.82)	<0.001
Random effect							
Variance		0.69	<0.001	0.51	<0.001	0.28	<0.001
ICC		0.17		0.13		0.08	
Deviance		75,205.44		74,704.66		75,010.76	
Reliability		0.769		0.705		0.572	

The data were analyzed by multilevel logistic regression. Values are presented as odds ratio (95% confidence interval). OR, odds ratio; CI, confidence interval.

**Table 6:** Multilevel analysis of students’ high-intensity physical activity performance (two days a week or less).

Model Parameter		Model 1 Null Model		Model 2 Unconditional Slope Model		Model 3 Final Model	
		OR (95%CI)	*p*	OR (95%CI)	*p*	OR (95%CI)	*p*
Fixed effect							
Level 1	Intercept γ_00_	2.79 (2.62–2.98)	<0.001	5.47 (4.82–6.21)	<0.001	6.97 (5.66–8.58)	<0.001
	Sex (boy vs. girl)			0.25 (0.23–0.28)	<0.001	0.25 (0.23–0.28)	<0.001
	Grade (1st vs. 3rd)			0.93 (0.85–1.02)	0.120	0.93 (0.85–1.02)	0.119
	Grade (2nd vs. 3rd)			0.90 (0.82–0.99)	0.030	0.90 (0.82–0.99)	0.029
	Self-reported economic status (lower vs. upper)			1.07 (0.98–1.18)	0.114	1.09 (1.00–1.19)	0.055
	Self-reported economic status (middle vs. upper)			1.17 (1.10–1.24)	<0.001	1.17 (1.11–1.25)	<0.001
	Academic performance (lower vs. upper)			0.75 (0.69–0.81)	<0.001	0.75 (0.69–0.81)	<0.001
	Academic performance (middle vs. upper)			0.93 (0.86–1.01)	0.070	0.93 (0.86–1.00)	0.059
	Self-reported health status (unhealthy vs. healthy)			2.07 (1.94–2.22)	<0.001	2.08 (1.94–2.23)	<0.001
	Self-reported oral health status (unhealthy vs. healthy)			-	-	-	-
	Experience of stress (yes vs. no)			-	-	-	-
	Experience of depression (yes vs. no)			-	-	-	-
	Oral symptoms (yes vs. no)			1.11 (1.05–1.17)	<0.001	1.10 (1.04–1.17)	<0.001
Level 2	School system (vocational vs. general)					0.80 (0.72–0.89)	<0.001
	School type (boys’ vs. co-ed)					1.14 (1.03–1.26)	0.013
	School type (girls’ vs. co-ed)					1.05 (0.90–1.23)	0.599
Random effect							
Variance		0.41	<0.001	0.14	<0.001	0.13	<0.001
ICC		0.11		0.04		0.04	
Deviance		77,688.94		77,807.54		77,805.90	
Reliability		0.826		0.611		0.593	

The data were analyzed by multilevel logistic regression. Values are presented as odds ratio (95% confidence interval). OR, odds ratio; CI, confidence interval.

## Data Availability

The data that support the findings of this study are available in Korea Disease Control and Prevention Agency at https://www.kdca go.kr/yhs/ (accessed on 1 December 2021).

## References

[1] Guariguata L., Jeyaseelan S. (2019). Children and Non-Communicable Disease: Global Burden Report 2019.

[2] Proimos J., Klein J.D. (2012). Noncommunicable diseases in children and adolescents. Pediatrics.

[3] NCD Alliance, FDI World Dental Federation (2020). Accelerating Action on Oral Health and NCDs: Achieving an Integrated Response.

[4] Centers for Disease Control and Prevention Trends in the Prevalence of Obesity and Dietary Behaviors National YRBS: 1991–2019. https://www.cdc.gov/healthyyouth/data/yrbs/factsheets/2019_obesity_trend_yrbs.htm.

[5] Centers for Disease Control and Prevention Nutrition, Physical Activity, and Obesity: Data, Trends and Maps. https://www.cdc.gov/nccdphp/dnpao/data-trends-maps/index.html.

[6] Substance Abuse and Mental Health Services Administration (2020). Key Substance Use and Mental Health Indicators in the United States: Results from the 2019 National Survey on Drug Use and Health (HHS Publication No. PEP20-07-01-001, NSDUH Series H-55).

[7] Ministry of Education, Ministry of Health and Welfare, Korea Disease Control and Prevention Agency (2019). The 15th Korean Youth Risk Behavior Survey.

[8] Viner R.M., Ozer E.M., Denny S., Marmot M., Resnick M., Fatusi A., Currie C. (2012). Adolescence and the social determinants of health. Lancet.

[9] De Coen V., Vansteelandt S., Maes L., Huybrechts I., De Bourdeaudhuij I., Vereecken C. (2012). Parental socioeconomic status and soft drink consumption of the child. The mediating proportion of parenting practices. Appetite.

[10] Kim A.Y., Kim J.H., Kye S.H. (2018). Sugar-sweetened beverage consumption and influencing factors in Korean adolescents: Based on the 2017 Korea youth risk behavior web-based survey. J. Nutr. Health.

[11] Park H.S., Kim N.H. (2008). Predicting factors of physical activity in adolescents: A systematic review. Asian Nurs. Res..

[12] Levin K.A., Currie C. (2010). Adolescent toothbrushing and the home environment: Sociodemographic factors, family relationships and mealtime routines and disorganisation. Community Dent. Oral Epidemiol..

[13] Commission on Social Determinants of Health (2008). Closing the Gap in a Generation: Health Equity through Action on the Social Determinants of Health.

[14] World Health Organization (2003). The World Oral Health Report 2003: Continuous Improvement of Oral Health in the 21st Century—The Approach of the WHO Global Oral Health Programme.

[15] Singh A., Bassi S., Nazar G.P., Saluja K., Park M., Kinra S., Arora M. (2017). Impact of school policies on non-communicable disease risk factors—A systematic review. BMC Public Health.

[16] Graham H., Power C. (2004). Childhood disadvantage and health inequalities: A framework for policy based on lifecourse research. Child Care Health Dev..

[17] Skara S., Sussman S., Dent C.W. (2001). Predicting regular cigarette use among continuation high school students. Am. J. Health Behav..

[18] Dahlui M., Jahan N.K., Majid H.A., Jalaludin M.Y., Murray L., Cantwell M., Su T.T., Al-Sadat N., MyHeARTs Group (2015). Risk and protective factors for cigarette use in young adolescents in a school setting: What could be done better?. PLoS ONE.

[19] Miller G.F., Sliwa S., Brener N.D., Park S., Merlo C.L. (2016). School district policies and adolescents’ soda consumption. J. Adolesc. Health.

[20] Maes L., Lievens J. (2003). Can the school make a difference? A multilevel analysis of adolescent risk and health behaviour. Soc. Sci. Med..

[21] World Health Organization (2013). Global Action Plan for the Prevention and Control of Noncommunicable Diseases 2013-2020.

[22] Fernandez de Grado G., Ehlinger V., Godeau E., Arnaud C., Nabet C., Benkirane-Jessel N., Musset A.M., Offner D. (2021). Changes in tooth brushing frequency and its associated factors from 2006 to 2014 among French adolescents: Results from three repeated cross sectional HBSC studies. PLoS ONE.

[23] Hanson M.D., Chen E. (2007). Socioeconomic status and health behaviors in adolescence: A review of the literature. J. Behav. Med..

[24] Fulkerson J.A., Sherwood N.E., Perry C.L., Neumark-Sztainer D., Story M. (2004). Depressive symptoms and adolescent eating and health behaviors: A multifaceted view in a population-based sample. Prev. Med..

[25] Ministry of Education Secondary Education. http://english.moe.go.kr/sub/info.do?m=020103&s=english.

[26] Reliable Ministry of Government Legislation Korean Law Information Center Enforcement Decree of the Elementary and Secondary Education Act. https://www.law.go.kr.

[27] Santelli J.S., Sivaramakrishnan K., Edelstein Z.R., Fried L.P. (2013). Adolescent risk-taking, cancer risk, and life course approaches to prevention. J. Adolesc. Health.

[28] Jung S.H., Tsakos G., Sheiham A., Ryu J.I., Watt R.G. (2010). Socio-economic status and oral health-related behaviours in Korean adolescents. Soc. Sci. Med..

[29] Beaglehole R., Bonita R., Horton R., Adams C., Alleyne G., Asaria P., Baugh V., Bekedam H., Billo N., Casswell S. (2011). Priority actions for the non-communicable disease crisis. Lancet.

[30] Kim S.I., Lee H.R., Ma D.S., Park D.Y., Jung S.H. (2012). The differences of oral health-related behaviors by type of school among high school students in Gangneung city. J. Korean Acad. Oral Health.

[31] Bae E.J., Yoon J.Y. (2019). Factors associated with current smoking among male high school students according to school type: Using data from the 13th (2017) Korea youth risk behavior web-based survey. J. Korean Soc. Sch. Health.

[32] Atorkey P., Byaruhanga J., Paul C., Wiggers J., Bonevski B., Tzelepis F. (2021). Multiple health risk factors in vocational education students: A systematic review. Int. J. Environ. Res. Public Health.

[33] World Health Organization (2020). Life Skills Education School Handbook Prevention of Noncommunicable Diseases.

[34] Busch V., De Leeuw R.J., Schrijvers A.J. (2013). Results of a multibehavioral health-promoting school pilot intervention in a Dutch secondary school. J. Adolesc. Health.

[35] Ministry of Education, Korea Educational Environments Protection Agency 10+ Growth Story of Health Promoting School in Korea. https://www.schoolhealth.kr/ebook/10th_gstory_eng/.

[36] Khoshnevisan M.H., Pakkhesal M., Jadidfard M.P., Godarzi Nejad G. (2019). School-based oral health promotion: A thorough review. J. Dent. Sch..

[37] Veugelers P.J., Schwartz M.E. (2010). Comprehensive school health in Canada. Can. J. Public Health.

